# From chill to bloom: seasonal coordination of reproductive development in peach and temperate fruit trees

**DOI:** 10.1093/jxb/erag128

**Published:** 2026-03-09

**Authors:** Serena Varotto, Donato Giannino, Claudio Bonghi

**Affiliations:** Department of Agronomy, Food, Natural Resources, Animals and Environment, University of Padova, Viale dell’Università 16, Legnaro (PD) 35020, Italy; Consiglio Nazionale delle Ricerche, Istituto per i Sistemi Biologici (ISB), Via Salaria Km. 29,300, Monterotondo Scalo, Roma 00015, Italy; Department of Agronomy, Food, Natural Resources, Animals and Environment, University of Padova, Viale dell’Università 16, Legnaro (PD) 35020, Italy; University College Dublin, Ireland

**Keywords:** Climate variability, dormancy, gametophyte development, megasporogenesis, microsporogenesis, seasonal temperature

## Abstract

Seasonal temperature is the primary environmental cue controlling reproductive development in temperate fruit trees, yet its role has largely been interpreted through dormancy-based models that view winter cold as a passive prerequisite for growth resumption. This review reassesses this framework by examining cold and warmth as sequential developmental signals acting during late flower development, after inflorescence meristem identity has been established. Integrating anatomical, cytological, and transcriptomic evidence, we show that reproductive development follows a biphasic thermal organization. Microsporogenesis can progress during winter under chilling temperatures in a species-dependent manner, with peach representing a clear case of cold-driven meiotic progression. In contrast, pollen maturation and female gametophyte development remain dependent on rising spring temperatures and occur within a narrow pre-bloom window across major *Rosaceae* fruit crops. We further discuss how this sequential thermal control is coordinated by multiple regulatory layers involving transcriptional regulation, hormonal balance, carbohydrate metabolism, and chromatin dynamics. Disruption of the sequence of cold and warm periods under recent climate variability uncouples male and female gametophyte development, leading to recurrent failure modes that compromise fertility. We conclude that winter represents an active developmental phase and that reproductive vulnerability arises primarily from altered thermal sequencing rather than cumulative temperature deficits.

## Teaser

Winter cold actively shapes reproductive development in fruit trees, advancing male meiosis while female gamete formation waits for spring, creating a fragile seasonal coordination increasingly disrupted by temperate climate perturbations.

## Introduction

Temperature is the dominant seasonal cue governing development in temperate fruit trees, yet its role in reproductive biology has long been interpreted through a largely permissive framework. For decades, flowering phenology has been inferred indirectly from dormancy-based models (Campoy *et al*., 2011), which describe dormancy as a state in which buds remain largely developmentally static until chilling requirements are fulfilled and subsequent heat accumulation allows growth to resume (Lang, 1987). Quantitative efforts have therefore focused on estimating hours of cold (temperatures below a species-specific threshold) and heat units (temperatures above a base value) required to reach blooming, usually defined as anthesis (Fadón *et al*., 2020). Within this classical view, cold is regarded merely as a prerequisite for the release of winter dormancy, while warmth is considered the true driver of visible growth and reproductive progression.

In the present review, we distinguish between visible growth resumption and developmental progression. Dormancy-based models traditionally interpret winter as a period lacking growth because organ expansion and external morphological change are absent. However, absence of visible growth does not imply absence of development. Instead, specific reproductive programmes may continue under low temperature through regulated cellular differentiation, meiotic progression, and transcriptional reprogramming. Throughout this review, winter is therefore not considered a permissive phase preceding development, but a developmental phase with its own temperature-dependent trajectory. Within this interpretation, chilling accumulation and heat requirements are not treated as interchangeable quantitative inputs but as sequential signals acting on distinct reproductive processes with different seasonal competencies.

From this perspective, seasonal temperature does not simply modulate the rate of a single developmental programme, but determines which reproductive processes are competent to proceed at a given time. Consequently, winter does not uniformly slow development: it selectively permits specific stages while preventing others. This differential seasonal competence generates temporal separation among reproductive processes, explaining why comparable cumulative chilling and heat accumulation can result in contrasting reproductive outcomes when their seasonal distribution differs.

Consistently, studies in woody species have shown that low temperature cannot be interpreted as an unambiguous non-permissive condition for reproductive developmental progression. Its developmental effect depends on the physiological dormancy status of the bud, allowing the same thermal regime to either maintain developmental arrest or permit ontogenetic progression (Lundell *et al*., 2020). In temperate fruit trees, this interaction becomes particularly evident during the autumn–winter period following the transition of the shoot apical meristem to reproductive fate, a phase in which the progression of reproductive structures under low temperature depends on the developmental state already achieved at the onset of the cold season (Koutinas *et al*., 2010). Together, these observations indicate that winter cold does not interact with developmental programmes in a single, conserved manner. As a result, reproductive development is guided by the timing and sequence of cold and warm periods, rather than solely by their cumulative effects.

Observations made under recent climate variability reinforce this reinterpretation by highlighting situations in which dormancy-based models fail to capture the effect of altered thermal sequences (Baumgarten *et al*., 2021). Several phenological and developmental studies have shown that aberrant winter and early-spring temperature patterns are associated with altered trajectories of reproductive development in fruit trees (El Yaacoubi *et al*., 2014; Kumar *et al*., 2024). Although species differ in the underlying mechanisms, these responses indicate that temperature anomalies expose windows of developmental receptiveness that remain masked under stable seasonal regimes.

In this review, by integrating anatomical, cytological, physiological, and transcriptomic evidence, we examine how cold and warmth act as sequential developmental signals during the late phases of reproductive development, after the identity of the inflorescence meristem has been established. This perspective provides a basis for reconsidering the classical chilling–heat model and for understanding how climate warming may affect the coordination of microsporogenesis and megasporogenesis, with consequences for fertility and yield stability.

## Rethinking seasonal control of reproductive development in temperate fruit trees

Differences in winter behaviour among temperate fruit tree species arise from the structural organization and the developmental state of buds entering the cold season. According to Rohde and Bhalerao (2007), the winter rest (dormancy) corresponds to ‘an inability to initiate growth under favourable conditions’, a definition that emphasizes the intrinsic developmental competence of the structures involved. Applying a generalized dormancy framework across species, without accounting for bud type and organ composition, therefore risks obscuring meaningful biological differences in how temperature interacts with reproductive development.

In temperate fruit trees, reproductive organs may develop within mixed buds, in which flower and vegetative primordia co-exist, or within flower buds containing only reproductive structures (Koutinas *et al*., 2010; Costes *et al*., 2014). This architectural distinction has direct developmental consequences. In mixed buds, the co-existence of vegetative and flower tissues gives rise to asynchronous developmental trajectories and differential responses to environmental cues (Bubán and Faust, 1982, 1995). Consequently, temperature signals, particularly during winter, tend to stabilize structures already differentiated during the warm season rather than to promote coordinated progression of the reproductive programme. By contrast, species that produce vegetative and flower buds as distinct structures enter winter with a more homogeneous meristematic identity within the flower buds, allowing temperature signals to be integrated more coherently within a single reproductive pathway. Insights from *Prunus persica* illustrate this pattern, as the anatomical separation of vegetative and flower buds makes it possible to disentangle their respective developmental trajectories.

Specifically, the peach bud system is useful for examining late meristem differentiation and fate over the entire course of flower development, without the confounding influence of concurrent vegetative development. Recent evidence shows that peach flower and vegetative buds share similar winter hormonal patterns but deploy distinct MADS-box combinations within an organ-specific DORMANCY-ASSOCIATED MADS-box (DAM)/SHORT VEGETATIVE PHASE (SVP) regulatory circuitry (Joseph *et al*., 2026). This prompts the idea that asynchrony in mixed buds of other species reflects intrinsic differences between vegetative and inflorescence meristems rather than their simple physical co-existence. Building on this distinction between bud types, peach offers a particularly informative case for understanding how temperature modulates reproductive development during winter. Anatomical studies have long demonstrated that peach flower buds continue to differentiate during the chilling period, progressing through clearly defined stages even at low temperatures (Luna *et al*., 1990; Reinoso *et al*., 2002; Giannino *et al*., 2003). Transcriptomic profiles reinforce this view: key gene networks related to chromatin dynamics, sugar mobilization, hormone biosynthesis and signalling, meiotic initiation, and stress adaptation remain active throughout the chill accumulation phase (Canton *et al*., 2022; Joseph *et al*., 2026). In this context, cold does not simply remove constraints on growth but contributes actively to shaping the reproductive trajectory.

These observations in peach challenge the classical chilling–heat model assumption that buds enter winter in comparable developmental states and that cold and warmth act on a uniform developmental framework. The influence of temperature on reproductive development is pre-conditioned by two axes that vary widely among species: (i) the ontogenetic stage reached by the reproductive bud at the onset of winter; and (ii) the intrinsic regulatory competence of the meristem, defined as its capacity to sustain transcriptional and metabolic programmes under low temperature. Together, these axes determine which components of the reproductive programme are available to respond to temperature decline and which remain dependent on subsequent warming. When these factors are considered explicitly, winter behaviour can be interpreted as a continuum of temperature–development relationships rather than as a binary dormant/non-dormant state. At one end of this continuum lies *P. persica*, in which reproductive buds enter winter with a well-defined flower architecture and retain the capacity for cold-driven progression. Consequently, low temperature directly contributes to advancing specific steps of reproductive development, supported by robust cold-responsive regulatory activity.

Other *Prunus* species, such as sweet cherry (*Prunus avium*) and apricot (*Prunus armeniaca*), occupy an intermediate position along this continuum. In these species, flower buds are distinct from vegetative buds and contain flower whorls (including ovary primordia) that are largely defined before winter, while showing limited morphological progression during the cold season (Julian *et al*., 2010; Fadón *et al*., 2018). Nevertheless, buds display measurable winter transcriptional and metabolic activity, indicating that cold engages regulatory processes even when overt structural advancement is constrained (Canton *et al*., 2021). This view is further supported by epigenomic and transcriptomic analyses in sweet cherry, showing that chilling accumulation is accompanied by extensive DNA methylation remodelling and coordinated transcriptional reprogramming during dormancy, well before resumption of visible growth (Rothkegel *et al*., 2020). Here, winter temperature appears to modulate developmental competence and timing, rather than directly driving organogenesis progression.

Further along the continuum, species such as apple (*Malus×domestica*) and kiwifruit (*Actinidia* spp.) enter winter with less advanced reproductive structures, and organogenesis remains largely static until warming resumes (Bubán and Faust, 1982; Walton *et al*., 1997; Wall *et al*., 2008; Koutinas *et al*., 2010). Molecular and regulatory studies nonetheless reveal selective winter activity associated with dormancy maintenance, competence acquisition, and preparation for reactivation, rather than with direct advancement of the reproductive programme (Mimida *et al*., 2015; Voogd *et al*., 2015; Chen *et al*., 2022; Wu *et al*., 2025). In these species, cold acts primarily on regulatory readiness rather than on developmental progression itself.

Taken together, these comparative observations show that the developmental impact of winter temperature is contingent on bud architecture and ontogenetic history, rather than acting as a generalized input across temperate fruit species. Which steps of the reproductive pathway can respond during winter, and which remain deferred until spring, is therefore determined by the developmental context established before winter.

Reframing winter in this way transforms the interpretation of seasonal control. Cold and warmth should not be treated as interchangeable contributors to a single chilling–heat balance, but as signals that act on distinct reproductive processes with different seasonal competencies, rather than on a unique developmental programme. This framework provides the conceptual foundation for the differentiated view of reproductive progression developed in the following sections, where the distinct thermal regulation of male and female gametophyte development is examined in detail.

## Cold-driven microsporogenesis as a defining feature of peach reproductive development

**Fig. 1.**
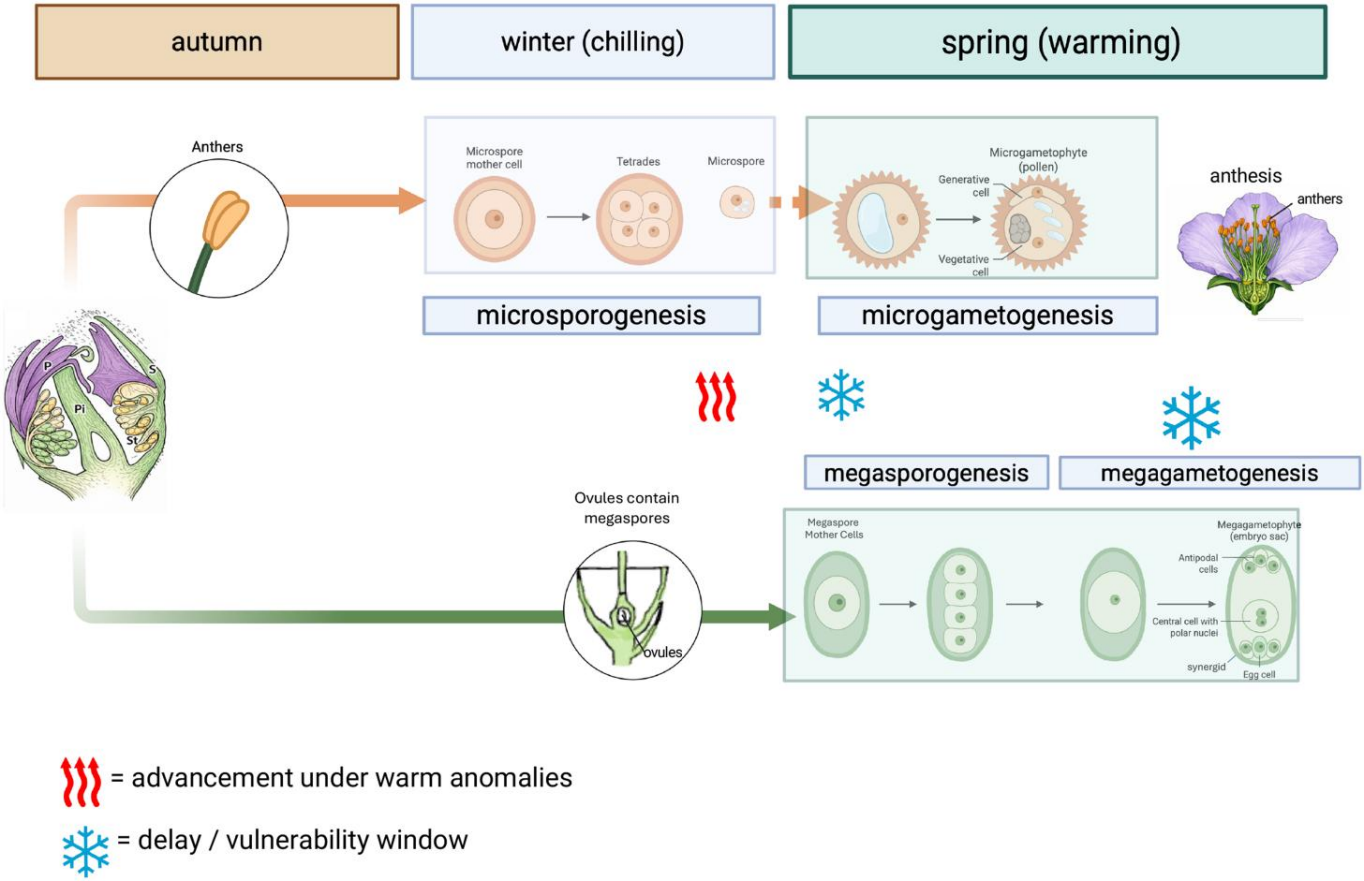
Biphasic organization of reproductive development in peach (*Prunus persica*). Schematic representation of the seasonal separation between male and female reproductive development in peach flower buds. During autumn and winter (chilling period), anther development proceeds under low temperatures and encompasses microsporogenesis, including meiotic divisions of pollen mother cells and the formation of free microspores. In peach, this phase can be completed before visible bud growth, with microspores showing fragmented vacuoles prior to their coalescence. As temperatures rise in spring, microgametogenesis resumes: the formation of a single large vacuole precedes the first pollen mitosis, leading to the asymmetric division that generates a large vegetative cell—characterized by prominent amyloplast accumulation—and a smaller generative cell, resulting in a binucleate pollen grain typical of *Prunus* species (Brewbaker, 1967). In contrast, female reproductive development remains largely quiescent during winter. In peach, ovule differentiation and embryo sac development are delayed until spring warming, with megasporogenesis and subsequent megagametogenesis occurring after dormancy release. The pistil is monocarpellate and contains two ovules, whose developmental progression is tightly linked to post-dormancy temperature conditions (Arbeloa and Herrero, 1991). Heat and snowflake symbols indicate differential sensitivity of male and female pathways to temperature anomalies, highlighting how warm winter events may advance male development, while delayed or unstable spring warming can generate vulnerability windows for female gametophyte development (see Table 1 for details). Created with BioRender, C. Bonghi (2026) https://BioRender.com/2u5ujob and further edited in Powerpoint.

Among temperate fruit species, *P. persica* provides a clear example of cold-mediated microsporogenesis, in which microspore and early pollen development events unfold during the cold season. Reproductive organs consist of the stamen, composed of a filament supporting the anther, where microsporogenesis occurs. In peach, stamen primordia and anther structures are initiated during the warm season, establishing the sporophytic framework before winter onset (Fig. 1). Pollen development is commonly divided into two sequential phases: microsporogenesis, which terminates when single microspores are released from the meiosis-derived tetrads; and microgametogenesis, which encompasses the progression from the first microspore mitosis leading to the formation of a bicellular pollen grain. In species producing binucleate pollen, such as peach, the second mitotic division of the generative cell occurs later during pollen tube growth (Brewbaker, 1967). Cytological studies of peach flower buds collected during late autumn, and winter (during chilling accumulation) reveal that meiosis in pollen mother cells (PMCs) takes place in fully enclosed flower buds, several weeks before flower opening. At the end of the chilling period, young pollen is present in anthers, indicating that microsporogenesis is completed well before flower opening and anthesis (i.e. before visible bud swelling) (Luna *et al*., 1990; Reinoso *et al*., 2002). These observations indicate that the initiation and completion of microsporogenesis are temporally aligned with the cold season itself, rather than with the resumption of visible growth.

Cytological analyses of anther development show that differentiation of the anther wall, encompassing the epidermis, endothecium, middle layer, and tapetum, as well as the early stages of microspore development, proceeds under chilling temperatures (Luna *et al*., 1990). Flower buds display continuous development from autumn to flowering, with androecial structures developing throughout the cold period, whereas gynoecial structures differentiate in late winter (Luna *et al*., 1990). As a result, in peach, microsporogenesis is completed before visible bud swelling and before the onset of female gametophyte differentiation, revealing a clear temporal dissociation between male and female reproductive development. Transcriptomic analyses in peach corroborate these findings. Canton *et al*. (2022) demonstrated that key meiotic genes, including homologues of *DMC1*, *RAD51*, and *ASY1*, are significantly up-regulated in peach flower buds during the cold period. These expression patterns correlate with the cytological evidence of ongoing meiosis and support the idea that chilling temperatures not only permit, but actively sustain, the initiation and progression of male gametophyte differentiation and gamete production. In parallel, genes related to chromatin remodelling, RNA processing, and hormonal signalling—notably gibberellins (GAs) and jasmonates—also exhibit winter-specific expression peaks, pointing to an orchestrated cold-mediated molecular programme underlying male reproductive development.

This developmental strategy, however, is not fully conserved across *Prunus* species. In apricot, anther development proceeds up to pre-meiotic stages during endodormancy (a phase in which buds are unable to resume growth even under otherwise favourable external conditions), while the onset of meiosis coincides with the fulfilment of chilling requirements and the subsequent dormancy transition (Julian *et al*., 2011, 2014). Multi-year field observations further support this interpretation, demonstrating that the timing of male meiosis closely tracks chilling accumulation and can be used as a biological marker of the endo- to ecodormancy transition (Herrera *et al*., 2022), which is the shift to a phase in which buds are competent to grow but remain quiescent because external conditions are still unfavourable (Lang, 1987). These observations indicate that, in apricot, cold primarily defines the temporal window in which microsporogenesis can occur, acting as a gate for meiotic competence rather than as a direct driver of continuous developmental progression. A comparable situation has been described in sweet cherry, where anther and pollen development are likewise constrained by dormancy dynamics. Cytological analyses demonstrate that male meiosis is strictly linked to the completion of chilling and is further modulated by subsequent forcing conditions (Fadón *et al*., 2019). Controlled forcing experiments confirmed that the progression of microsporogenesis depends on the sequential fulfilment of chilling and warm temperature requirements, highlighting that cold alone is insufficient to sustain male meiotic development in this species (Fadón *et al*., 2021). Together, these findings indicate that, unlike peach, sweet cherry relies on a cold–warmth sequence in which winter temperatures establish meiotic competence, while developmental progression requires the onset of favourable thermal conditions.

Transcriptomic evidence supports this interpretation by revealing fundamental differences in the temporal coupling between cold exposure and male meiotic activation among *Prunus* species. A meta-analysis of RNA-seq studies on flower buds of sweet cherry, apricot, and peach showed that genes associated with PMC meiosis and early pollen development are not uniformly activated throughout winter, but display species-specific timing patterns linked to dormancy progression. In both apricot and sweet cherry, meiotic and pollen-related genes are preferentially up-regulated towards the end of endodormancy or during the transition to ecodormancy, consistent with cytological evidence indicating that cold primarily establishes meiotic competence rather than sustaining continuous microsporogenesis. Temporal differences in gene expression patterns were associated with differences in the chilling requirements of analysed genotypes in the three species (Canton *et al*., 2021). Evidence from almond (*Prunus dulcis*) further supports this intermediate scenario. Studies combining phenological, anatomical, and physiological analyses indicate that male reproductive development is tightly coupled to dormancy progression and is highly sensitive to thermal conditions around dormancy release, with cold contributing to the acquisition of developmental competence and subsequent warming required for proper progression of pollen development (Barros *et al*., 2012; El Yaacoubi *et al*., 2016; Keleta *et al*., 2023).

*Actinidia* provides a contrasting case that further sharpens the interpretation of peach. In *A. arguta*, detailed cytological analyses (Li *et al*., 2024) describe a complete sequence of male and female gametogenic events but do not associate them with winter progression. Earlier work in kiwi from other groups (Wall *et al*., 2008) demonstrated that stamen and pistil organogenesis occur only after chilling requirements have been fulfilled and growth has resumed under warmer conditions. Unlike peach, *Actinidia* does not show evidence of microsporogenesis advancing during cold accumulation; rather, cold acts primarily as a permissive factor that enables development to initiate later. Including *Actinidia* in the comparison therefore highlights that cold-driven microsporogenesis is not a universal feature of temperate fruit species and that peach represents a distinct reproductive strategy in which the male lineage progresses during the chilling period itself.

Although microsporogenesis can be completed under winter conditions, the subsequent phase of microgametogenesis—from the first pollen mitosis to pollen maturation—does not progress during chilling and requires rising temperatures after dormancy release, as indicated by cytological observations in peach and by forcing experiments in other *Prunus* species (Reinoso *et al*., 2002; Fadón *et al*., 2019, 2021).

Taken together, these observations indicate that microsporogenesis represents the first step of gametophyte development subjected to seasonal temperature regulation. Across *Prunus* species, winter does not constitute a passive interval for the male reproductive structures but defines a critical temporal framework in which meiotic competence is established and, in some cases, actively exploited for the completion of meiosis. Cold temperatures sustain microsporogenesis in a species-dependent manner, creating a seasonal asymmetry in reproductive development: microsporogenesis is anchored to winter conditions, whereas microgametogenesis and female gametophyte development depend on rising spring temperatures. Seasonal temperature therefore does not act as a uniform regulator of reproductive development but selectively influences the different reproductive structures.

## Female gametogenesis in temperate fruit trees: developmental foundations and thermal sensitivity

Briefly, the female reproductive organ (pistil) produces a diploid megaspore mother cell (MMC), which undergoes meiosis (megasporogenesis) from which haploid megaspores develop into the female gametophyte (embryo sac containing the egg and accessory cells). Despite its central role in sexual reproduction, female gametophyte development remains one of the least documented processes in temperate fruit trees. Detailed cytological descriptions of the complete sequence from archesporial cell specification to embryo sac maturation are available for only a limited number of species, most notably *A. arguta*, for which a complete sequence has recently been provided (Li *et al*., 2024), whereas information for *Prunus* and other *Rosaceae* is fragmentary and often restricted to isolated developmental stages or focuses on ovule viability (Arbeloa and Herrero, 1991; Nava *et al*., 2009; Đorđević *et al*., 2024; Erfatpour *et al*., 2024).

A comparative perspective nevertheless reveals a coherent pattern. *Actinidia* enters winter with an undifferentiated or only partially defined flower meristem (Walton *et al*., 1997), and neither megasporogenesis nor megagametogenesis progresses during the cold season; MMC differentiation and meiosis occur only after chilling fulfilment, under rising spring temperatures (Wall *et al*., 2008), as confirmed by detailed cytological analysis (Li *et al*., 2024). In peach, by contrast, flower buds at the onset of the cold season contain ovular primordia morphologically well defined (Reinoso *et al*., 2002; Giannino *et al*., 2003). However, despite this more advanced anatomical state relative to *Actinidia*, MMC appearance and entry into meiosis remain confined to the immediate pre-bloom phase (Fig. 1), indicating that female meiotic competence is not acquired during the cold season. This pattern is not unique to peach but recurs across multiple *Prunus* species—including apricot (Alburquerque *et al*., 2002), Japanese plum (Ruiz *et al*., 2010), almond (Cousin and El Maataoui, 1998), and both sweet and sour cherry (Chudíková *et al*., 2012), all showing that MMC specification and female meiosis require warming conditions, irrespective of the developmental stage attained by the ovule before winter. This convergence indicates that female gamete development in temperate fruit trees is primarily constrained by intrinsic thermal thresholds governing the acquisition of developmental competence, rather than by the anatomical stage reached before winter. Subsequently, progression and functionality depend on adequate metabolic support, including localized sugar allocation (Singh *et al*., 2023) and sink activity within reproductive and vegetative organs (Falchi *et al*., 2020). Molecular insights from model systems reinforce this interpretation. The regulatory networks that specify sporogenous cell identity, initiate meiosis, and orchestrate early megagametogenesis, as described in model species (Kaur *et al*., 2024), are not associated with evident activation of the female meiotic programme under winter low-temperature conditions in temperate trees, consistent with the confinement of MMC differentiation and female meiosis to the pre-bloom warming window. The female gamete formation emerges as a heat-dependent module, activated only when temperatures rise in the pre-bloom phase, even in species that maintain other forms of flower progression during winter.

In temperate fruit trees, because activation occurs only shortly before bloom, the female pathway encounters a second intrinsic constraint: the embryo sac remains structurally and physiologically intact only for a limited period. Detailed cytological analyses in almond and apricot demonstrate that a fully developed embryo sac is present only in the days immediately preceding bloom (Cousin and El Maataoui, 1998; Alburquerque *et al*., 2002), leaving a narrow interval for successful fertilization. Comparable observations in pear reveal a similar pattern, with ovule degeneration occurring soon after megagametogenesis and closely linked to the timing of embryo sac formation (Guerra *et al*., 2011; Cerović *et al*., 2020). Even in cold-climate environments, delayed completion of embryo sac development restricts the period during which the ovule remains structurally viable (Cerović *et al*., 2022).

In summary, for *Prunus* spp., regardless of how far ovular primordia have differentiated before winter, MMC appearance, meiosis, and embryo sac maturation occur in the warm pre-bloom window; these observations reveal a reproductive system structured around an asymmetric thermal logic. Male gametophyte development, which can continue its trajectory under winter cold, occupies a broad temporal window. The female reproductive pathway, by contrast, acquires competence only under rising temperatures in the immediate pre-bloom phase, within a narrow temporal window.

Reproductive success therefore depends on the alignment of two sequential modules—a cold-responsive male pathway and a heat-dependent female pathway—whose coordination is achieved rather than pre-determined and remains intrinsically sensitive to temperature. Under historically stable climates this synchronization was generally maintained; under current patterns of winter and early-spring variability, the narrow thermal window that governs the female lineage emerges as the principal point of vulnerability.

## Temperature anomalies and the disruption of thermally guided reproductive development

Thermal anomalies, whether expressed as winter warming, spring cooling, or rapid oscillations between cold and warm conditions, destabilize the seasonal organization on which reproductive development normally relies. Their effect is not a direct physiological stress on flower tissues, but a shift, and in some cases an inversion, in the timing at which cold-responsive and heat-dependent phases are activated. This altered timing exposes the intrinsic fragility of a system that depends on the orderly progression from winter to spring (Guy, 2014; Cook *et al*., 2017; Fadón *et al*., 2020; Hartmann *et al*., 2024). To provide a structured overview of how distinct thermal anomalies disrupt the seasonal coordination of male and female reproductive development, the main recurrent failure modes described across temperate fruit trees are summarized in Table 1.

**Table 1.** Climate-related perturbations of male and female gametophyte development in *Rosaceae* fruit trees

Climatic anomaly type	Species	Bud type	Reproductive process affected	Observed alteration	Mechanistic interpretation	Reference
Mid-winter warm spell	*Prunus persica* (peach)	Flower bud	Microsporogenesis	Premature meiotic entry; advanced tetrad stage	Winter chilling establishes meiotic competence, enabling warm-induced advancement of microsporogenesis once forcing temperatures occur	Fadón *et al*. (2021); Szalay *et al*. (2024)
Mid-winter warm spell followed by renewed cooling	*Prunus persica*	Flower bud	Post-meiotic pollen development	Irregular tetrads; disrupted microspore cellularization	Warm-triggered progression exposes post-meiotic stages to subsequent cold, leading to structural instability during microspore development	Penso *et al*. (2020)
Insufficient winter chilling	*Prunus avium* (sweet cherry)	Flower bud	Microsporogenesis	Delayed or incomplete meiosis	Chilling establishes developmental competence but does not sustain meiotic progression in the absence of adequate forcing conditions	Fadón *et al*. (2019, 2021)
Cool pre-bloom temperatures	*Prunus armeniaca* (apricot)	Flower bud	Megasporogenesis/megagametogenesis	Delayed embryo sac maturation; reduced ovule viability	Female gametophyte development requires higher and more sustained thermal inputs than male development	Alburquerque *et al*. (2002); Nava *et al*. (2009)
Recurrent warm–cold oscillations	*Prunus avium*	Flower bud	Male–female developmental synchrony	Asynchrony between pollen and embryo sac development	Repeated activation and deactivation of temperature-sensitive developmental checkpoints disrupt coordinated progression of male and female pathways	Zhang *et al*. (2018); Szalay *et al*. (2024)
Prolonged cool spring	*Pyrus communis* (pear)	Mixed bud	Megagametogenesis	Incomplete embryo sac development; ovule degeneration	Female gametophyte viability is constrained within a narrow temporal and thermal window that is shortened under cool spring conditions	Cerović *et al*. (2020, 2022)
Short and fragmented warm phases	*Prunus* spp.	Flower bud	Female gametophyte development	Failure to complete embryo sac maturation	Compression of an already heat-dependent phase limits completion of megagametogenesis under unstable thermal regimes	Erfatpour *et al*. (2024)

The table summarizes documented effects of anomalous seasonal temperature patterns on microsporogenesis, microgametogenesis, megasporogenesis, and megagametogenesis across temperate fruit tree species. For each case, the affected reproductive process, the associated developmental alteration, and a mechanistic interpretation are reported based on anatomical, phenological, and cytological evidence. Interpretations reflect current physiological frameworks in which winter chilling establishes developmental competence, while subsequent warming enables or constrains progression, rather than direct molecular causality. Together, these observations illustrate how altered timing, duration, and sequencing of cold and warm periods can disrupt reproductive coordination, particularly under climate variability.

When winter includes intermittent warm episodes, microsporogenesis is often the first developmental component to shift, because its progression becomes responsive once part of the chilling requirement has been satisfied. Under these conditions, even brief mid-winter warm periods can trigger premature entry into meiosis or advance the tetrad stage, moving the anther into a developmental phase which is no longer coordinated with other flower organs or with the metabolic and hormonal state of the bud as a whole, and misaligned with the timing of female gametophyte development (Fadón *et al*., 2021; Szalay *et al*., 2024). This advancement becomes particularly vulnerable when temperatures drop again: the problem is not direct inhibition by cold, but that the warm signal has already moved microsporogenesis into a stage whose downstream dynamics are incompatible with renewed low temperatures. Consistent with this, cold exposure after premature advancement frequently disrupts post-meiotic organization (e.g. tetrad separation, vacuolization, and microspore cellularization), a pattern reported in *Prunus* species under low-chill conditions (Penso *et al*., 2020). This response fits well with field observations showing that warm interruptions accelerate the transition into heat-responsive phases even when chill accumulation is incomplete (Cook *et al*., 2017; Hartmann *et al*., 2024). Taken together, these findings indicate that warm spells act as active temporal cues, advancing microsporogenesis once meiotic competence has been established during chilling and thereby exposing post-meiotic stages to subsequent cooling without ensuring completion of downstream developmental processes.

The opposite situation arises under cool spells in the immediate pre-bloom period, when megagametogenesis becomes the limiting phase of flower development. Under such conditions, the organization of the embryo sac is markedly delayed relative to the external appearance of the flower (Alburquerque *et al*., 2002; Nava *et al*., 2009). Evidence from colder production areas reinforces this pattern: delayed or incomplete embryo sac maturation has been documented across plum and pear cultivars (Cerović *et al*., 2020, 2022), indicating that ovule development proceeds only within a narrow thermal window. Comparative developmental studies further support this interpretation, consistently showing that female gametophyte development exhibits higher thermal thresholds and a more compressed period of functional viability than other flower structures (Rodrigo *et al*., 2006; Hillmann *et al*., 2021). These delays do not represent ‘cold stress’ *sensu stricto*, but rather a reversion to a thermally incompetent state. Once temperatures fall below the activation threshold, megasporogenesis cannot resume, and even brief cool episodes are sufficient to push embryo sac development out of alignment with the rest of the flower.

The most destabilizing conditions arise when temperatures oscillate repeatedly between warm and cold. Such fluctuations impose incompatible demands on the two gametogenic sequences: microsporogenesis responds to warm pulses by advancing meiosis or progressing toward the tetrad stage, whereas female gametogenic sequence reverts to pre-meiotic states when temperatures drop again (Fadón *et al*., 2021; Szalay *et al*., 2024). Experimental and field observations corroborate this pattern. In sweet cherry and other *Prunus* species, oscillating temperatures increase the incidence of developmental irregularities, including reduced pistil viability and failures in embryo sac organization (Zhang *et al*., 2018). In apricot and apple, warm spells followed by sharp cooling generate buds in which externally advanced flower tissues co-exist with gynoecial structures showing microlesions or incomplete differentiation (Oukabli *et al*., 2003; Penso *et al*., 2020). These mixed anatomical states illustrate a central point: synchrony does not arise from a single linear progression but from the coordinated timing of development under stable thermal cues. Temperature oscillations disrupt this coordination more severely than persistent warming or cooling because they repeatedly trigger and suppress thermal checkpoints that the two developmental systems interpret differently (Szalay *et al*., 2024).

Thermal instability also leaves a recognizable anatomical footprint. Studies across *Prunus* and *Malus* species describe ovules with incomplete or degenerating embryo sacs, disorganized nucellar tissue, or sporogenous tissues arrested in late pre-meiotic stages under suboptimal temperatures (Nava *et al*., 2009; Cerović *et al*., 2020, 2022). Occasional cases of multiple MMC-like initials further indicate instability in early sporogenous identity (Chudíková *et al*., 2012). Parallel observations in the anther reveal irregular tetrads, abnormal cytokinesis, and binucleated microspores, particularly when warm pulses are followed by cooling (Đorđević *et al*., 2024).

All these conditions can be summarized into a small number of recurrent ‘failure modes’ that describe how temperature anomalies destabilize the biphasic organization of reproductive development. Warm winters favour an anticipation failure, in which microsporogenesis advances prematurely while female gametophyte development remains unresponsive (Fadón *et al*., 2021; Hartmann *et al*., 2024). Cool pre-bloom periods lead to a delay failure, where the acquisition of gametophytic competence occurs too late relative to the progression of microsporogenesis (Nava *et al*., 2009; Cerović *et al*., 2022). Rapid oscillations generate an oscillation failure, marked by repeated activation and deactivation of incompatible checkpoints and by the cytological abnormalities that accompany such reversals (Oukabli *et al*., 2003; Zhang *et al*., 2018; Penso *et al*., 2020). Finally, years with short and fragmented warm phases give rise to a compression failure, an emerging but less-documented phenomenon, in which the already narrow window for female gametophyte development becomes too short to be completed reliably (Cerović *et al*., 2020; Erfatpour *et al*., 2024). These failure modes are not mutually exclusive: they represent recurring expression of a single principle; temperature anomalies alter the seasonal phasing of distinct developmental programmes rather than imposing generic heat or cold stress.

Climatological analyses across major fruit-producing regions confirm that the thermal patterns described above are no longer rare episodes but rather increasingly frequent components of present-day seasons. Mild winters with reduced chill accumulation, fragmented warm phases, and recurrent warm–cold oscillations have been documented consistently over the past decades (Darbyshire *et al*., 2017; Fraga and Santos, 2021; Salama *et al*., 2021). As these anomalies shift from occasional events to structural features of regional climates, the developmental vulnerabilities outlined in this section—from the premature advancement of microsporogenesis to delays or incomplete progression of the female gametophyte—are expected to manifest with increasing regularity. Long-term datasets further show a progressive erosion of effective winter chill and an increase in intra-seasonal thermal variability (Noguer *et al*., 2023), providing the climatic context in which these reproductive misalignments are becoming systemic rather than episodic.

## Regulatory coordination of reproductive development under seasonal temperature cues

Controlling factors that are known to act on bud development during winter generally include hormones, carbohydrate metabolism, and oxidative stress (Beauvieux *et al*., 2018). Many recent reviews are available on the endogenous factors that influence the development of fruit tree buds and bud break during winter and early spring (Yang *et al*., 2021; Ding *et al*., 2024). Therefore, here we only intend to highlight what is currently known about these factors in relation to flower organ, and male and female gametophyte development in wintertime and the pre-bloom period.

The plant hormones abscisic acid (ABA) and GAs are among the most extensively studied endogenous regulators of bud dormancy in woody perennials (Liu and Sherif, 2019). These two hormones act antagonistically to control the induction, maintenance, and release of dormancy: endogenous ABA levels (and potentially ABA signalling) typically increase during autumn and winter and decline towards early spring, while GA levels show an opposite seasonal trend. Indeed, bud behaviour during winter has been conceptually compared with that of dormant seeds and vernalization, particularly with respect to the balance between the ABA and GA ratio. In the bud as a whole, the ABA and GA ratio remains stable during the wintertime (Yang *et al*., 2021) while no information is available for this hormone balance in the individual whorl. Cytokinin (CK) response regulators, which are important transcription factors for the modulation of CK-responsive genes, are putatively enrolled in spring growth of apple buds (Cattani *et al*., 2020). Beyond this core hormonal framework established in woody perennials, insights from model systems provide additional clues on hormonal control of gametophyte development. In Arabidopsis, brassinosteroids (BRs) play a central role in male reproductive development, regulating anther differentiation, tapetum function, pollen maturation, and fertility through tightly controlled spatial and temporal signalling (Li and He, 2020). Similarly, auxin has been shown to control key steps of female gametophyte development, including sporocyte specification and ovule patterning, through localized auxin maxima and downstream transcriptional networks (Cavalleri *et al*., 2025). While direct evidence for comparable roles of BRs and auxin during winter or pre-bloom stages in temperate fruit trees is still lacking, the conservation of these hormonal pathways suggests that they may contribute to fine-tuning gametophyte development once developmental competence has been established.

Carbohydrate metabolism is a key process during winter development, particularly in the final phases (Ito *et al*., 2013). Galactinol and raffinose are involved in a set of adaptive mechanisms acting synergistically to protect buds from the intrinsically limited water availability characteristic of winter conditions. Furthermore, the decline in the levels of these carbohydrates prior to budbreak suggests their mobilization as energy sources, in agreement with previous observations reported for other sugars (Zhuang *et al*., 2015; Hussain *et al*., 2016). A detailed analysis of starch accumulation patterns in sweet cherry flower buds revealed that they accumulated starch throughout the winter, with important variation in the starch content of the ovary primordia cells. While starch accumulated following a pattern similar to chilling accumulation, subsequently it vanished concomitantly with ovary development before budbreak (Fadón *et al*., 2018). These dynamics indicate that carbohydrate metabolism during winter is not merely protective but contributes to the energetic and developmental pre-conditioning of reproductive tissues.

A pivotal polymeric carbohydrate involved in the regulation of bud development and growth is callose, which is composed of β-1,3-glucans. Its deposition at plasmodesmata (PDs) is mediated by callose synthase genes (*CALS*/Glucan Synthase-Like genes), modulates symplastic connectivity, and thereby restricts the intercellular movement of macromolecules, including transcription factors (Hsieh *et al*., 2024). In woody perennials, callose accumulation at PDs has been linked to dormancy induction: in hybrid poplar, short days activate the ABA pathway, which promotes the expression of *CALS1* and represses glucanases that conversely degrade callose. The blockage of PDs is thought to restrict the bud from access to growth-promoting signals and favour cessation of bud growth (Singh *et al*., 2019). However, the high levels of *CALS* transcripts detected in reproductive tissues are consistent with the callose deposition observed during reproductive development in Arabidopsis and other plant species.

Callose deposition and *CALS* up-regulation have been observed in peach flower bud during chilling accumulation and flower differentiation (Canton *et al*., 2022). Moreover, transgenic apple lines overexpressing *PpDAM6*, encoding a transcription factor that is up-regulated in flower bud during winter, show increased callose deposition in the shoot apices, suggesting that *PpDAM6* might play a crucial role in cell–cell communication by regulating callose deposition (Zhao *et al*., 2023). In peach, callose patterning appears to be controlled by distinct genetic programmes in vegetative and floral buds, pointing to a context-dependent regulation of symplastic connectivity (Joseph *et al*., 2026).

Accumulating evidence has suggested that the bud redox status resulting from the balance between generation and scavenging of reactive oxygen species (ROS) plays a critical role in bud burst after cold development. As an important form of ROS, H_2_O_2_ has been considered as a key signalling molecule associated with dormancy release. The characteristics of H_2_O_2_ that make it a potential molecule marker for chilling accumulation and winter development include membrane permeability, relatively long lifetime, and oxidization of the critical thiol groups in some redox-sensitive proteins (Hubmann *et al*., 2022), which can theoretically lead to critical functional changes in proteins and transcription factors associated with development in wintertime. In addition, H_2_O_2_ is implicated in the control of the conductivity of PDs (Sager and Lee, 2014). The peak of H_2_O_2_ marks the achievement of chilling requirements or the transition to another phase, reinforcing the notion that H_2_O_2_, either as a signalling molecule and/or as a metabolite, could serve as a molecular marker for the progression of cold development and the acquisition of seasonal competence in deciduous woody perennials.

A fundamental question raised by the numerous studies in *Rosaceae* fruit trees concerns how the gene expression of the flower tissues embedded within the bud can be orchestrated in such a way to coordinately respond to endogenous and environmental signals.

A detailed analysis of flower development from initiation until bloom for early- and late-blooming sour cherries (*Prunus cerasus*) population segregating for a major bloom-time quantitative trait locus (QTL) showed that differences in the timing of microsporogenesis and megasporogenesis, as well as a coordinated maturation of male and female gametophytes, arise well before anthesis. Transcriptome analyses further identified numerous QTL-associated genes that are differentially expressed during the vegetative-to-floral transition. Importantly, the authors argued that comparisons starting at dormancy may obscure key developmental events, as both anatomical and molecular divergence were already detectable at early stages of floral initiation (Goeckeritz *et al*., 2024).

In this context, a comprehensive review by da Silveira Falavigna *et al*. (2019) highlighted that the regulatory module composed of *SVP* and *DAM* genes represents a conserved molecular framework linking early developmental trajectories with seasonal temperature cues in woody perennials. Across diverse tree species, a prominent functional orthologue of *SVP* is expressed at the onset of the cold season. In Arabidopsis, SVP is a MADS-box transcription factor acting as a floral repressor, directly binding and repressing the florigen gene *FLOWERING LOCUS T* (*FT*), with temperature modulating protein stability rather than transcript abundance (Lee *et al*., 2013).

Genomic surveys in *Rosaceae* have shown that SVP is represented by one orthologue in sweet cherry, and by two orthologues each in peach, apricot, and apple. In addition, studies in *P. persica* identified the activity of a set of *DAM* genes, which are phylogenetically related to *AGAMOUS-like* (*AGL24*) and *SVP*. In *Rosaceae*, *DAM* and *SVP* transcripts accumulated differentially in buds during winter, and their down-regulation is associated with bud burst. Consistently, QTLs controlling dormancy-related traits co-localize with *DAM* loci across multiple fruit trees species, including peach, apple, pear (*Pyrus communis*), Japanese apricot (*Prunus mume*), apricot, and sweet cherry (da Silveira Falavigna *et al*., 2019; Cantin *et al*., 2020).

Early studies in peach indicated that the expression of *DAM* genes was higher in cultivars with a high chilling requirement prior to chilling fulfilment, while it decreased in all cultivar types when bud break competence was reached (Jiménez *et al*., 2010). A subsequent meta-analysis on genes expressed during flower bud development showed that both *DAM* and *SVP* transcripts are up-regulated in autumn and strongly down-regulated during winter, in both HCR and LCR (high and low chilling requirement) genotypes of peach, sweet cherry, and apricot, while also revealing species-specific subfunctionalization among *DAM* paralogues (Canton *et al*., 2021). Functional studies further indicate that *DAM* activity depends on protein interactions with other MADS-box-like partners: in apple, overexpression of *MdDAMb* and *MdSVPa* delayed bud break (Wu *et al*., 2017) and the encoded proteins formed interacting complexes proposed to integrate environmental signals and restrict bud growth during winter dormancy (da Silveira Falavigna *et al*., 2021).

In plants, cold response studies have led to the identification of the C-REPEAT (CRT)/DEHYDRATION-RESPONSIVE ELEMENT (DRE) BINDING FACTORs (CBFs) and their encoding genes, which function as signalling hubs for cold acclimation (Ding *et al*., 2019). In turn, CBFs activate the expression of cold-regulated (*COR*) genes by binding to CRT/DREs in their promoters. During autumn, low non-freezing temperature is the second factor that, together with short daylength, regulates cold acclimation in fruit tree buds. Gene expression investigations on fruit tree bud development in early autumn have identified the fruit tree orthologues of CBF/DRE-binding (DREB), which are rapidly transcribed in response to cold and can induce the expression of *COR* genes. The latter are probably regulators of *DAM* gene transcription in response to cold (Akhtar *et al*., 2012; Yang *et al*., 2021). Indeed, peach *CBF1* overexpression in apple caused short-day-induced dormancy and significantly increased freezing tolerance (Wisniewski *et al*., 2015). Additionally in Japanese pear, promoter *cis*-elements analyses predicted the presence of a CBF-binding site (C-repeat/DRE element) in *ProDAM1* and *ProDAM3*, but not in *ProDAM2*. Yeast one-hybrid assays and transient assays validated the interaction between *PpCBF* and *PpDAM* (Niu *et al*., 2016). Thus, the presence of CBF-binding sites in DAM promoters might connect cold adaptation and bud winter development in *Rosaceae* fruit trees.

Epigenetic regulation represents a further mechanism through which the expression of *DAM* genes is controlled. In sweet cherry, higher levels of DNA methylation were found in the promoters of *PavMADS1* and *PavMADS2* after fulfilment of chilling requirements (Rothkegel *et al*., 2017). An increase in the abundance of siRNAs was associated with the observed *de novo* DNA methylation in the promoter region of *PavMADS1* (Rothkegel *et al*., 2017). DNA methylation and siRNAs are related to transcription repression when present at the promoter region, which suggests that these epigenetic changes modulate the down-regulation of *DAM* genes during dormancy in sweet cherry. Studies in *Prunus* and other fruit tree species have shown that *DAM* loci undergo complex chromatin regulation during bud winter development. In peach, *PpeDAM6* displays enrichment in H3K4me3 and H3K27me3 in promoter and intronic regions, accompanied by a decrease in H3Ac near dormancy release. In sweet cherry, H3K4me3 enrichment at *PavDAM5* correlates positively with the gene transcript levels. Further studies in peach and apple confirmed that H3K4me3 contributes to permissive chromatin states at *DAM* and *SVP* loci, whereas H3K27me3 does not consistently track changes in gene expression during chilling accumulation. Interestingly, other targets of chromatin modifiers were identified in pear and peach, where *NCED* and *GA2OX1* transcript accumulation is associated with H3K4me3 enrichment at the respective genomic loci (Canton *et al*., 2022; Yang *et al*., 2023). A significant correlation between H3K4me3 marks and gene expression of *NAC88*, *DAM* genes, and *CYC-J18* during pear bud cold development was also documented (Gao *et al*., 2021).

**Fig. 2.**
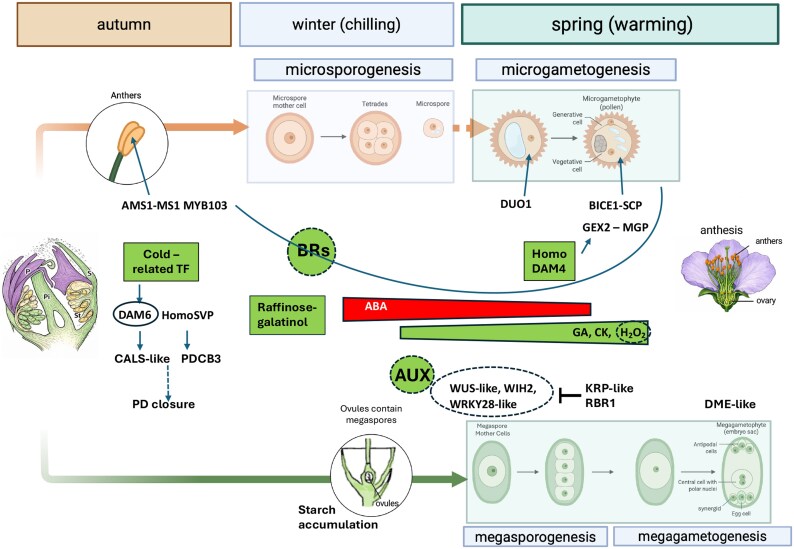
Schematic overview of the multilayer regulatory framework underlying male and female gametophyte development in *Prunus* species. Upper panel (male gametophyte development). Meta-analyses across *Prunus* species indicate that several tapetum- and pollen-related genes, including AMS1/MS1, MYB103, BICE/SCP, and DUO1, are expressed from the early phases of male gametophyte development, with expression already detectable during winter dormancy (Canton *et al*., 2021, see references therein). Transcriptomic and gene-regulatory evidence further indicates that DAM4 homodimers may target a GEX2–MGP gene, involved in late male gamete functionality in Arabidopsis (Mori *et al*., 2014), with significant transcript accumulation already during winter in peach flower buds (Joseph *et al*., 2026). These observations support the view that transcriptional programmes associated with male gametophyte competence are established before overt microgametogenic progression. Brassinosteroids have been implicated in the transcriptional regulation of pollen-related genes in model plants (Li and He, 2020). Lower panel (female gametophyte development). Meta-analyses across *Prunus* species (Canton *et al*., 2021, see references therein) show that genes involved in megaspore mother cell (MMC) specification (WUSCHEL-like, WINDHOSE2-like) and in the spatial restriction of sporogenous fate (WRKY28-like) are differentially and stage-specifically expressed during the pre-bloom phase. Entry of the MMC into meiosis is facilitated by the repression of WUS activity through KIP-RELATED PROTEIN (KRP) and RETINOBLASTOMA-RELATED1 (RBR1). The specification and maintenance of a single functional MMC are under epigenetic control (highlighted by an oval), involving components of the RNA-directed DNA methylation (RdDM) pathway and auxin-dependent processes (Cavalleri *et al*., 2025). In addition, DNA glycosylase DME-like transcripts are regulated during this period, consistent with imprinting and epigenetic reprogramming processes associated with the final phases of female gametophyte development. Regulatory layers (central). Winter chilling establishes lineage-specific developmental competence through the integration of multiple regulatory layers, including: (i) cold-responsive transcriptional regulation, mediated by CBF/DREB-like factors and their interaction with *DAM* genes (Wisniewski *et al*., 2015); (ii) whole-bud hormonal dynamics characterized by elevated ABA during winter and increasing GA, cytokinins, and H_2_O_2_ towards spring, while hormone dynamics at the level of individual floral whorls remain unresolved (Liu and Sherif, 2019; Cattani *et al*., 2020; Hubmann *et al*., 2022); (iii) carbohydrate metabolism, including raffinose and galactinol accumulation during winter and starch storage in ovary tissues, followed by pre-bloom mobilization (Fadón *et al*., 2018); (iv) distinct patterns of callose deposition observed in vegetative and flower buds during winter development under DAM/SVP-dependent regulation. *DAM* loci themselves are subject to epigenetic regulation (Canton *et al*., 2022; Joseph *et al*., 2026). Dotted lines indicate evidence derived from model plants. Created with BioRender. C. Bonghi (2026) https://BioRender.com/2u5ujob9, and further edited in Powerpoint.

Overall, at the molecular level, the construction of winter competence is accompanied by lineage-specific transcriptional priming, which precedes visible gametogenesis progression and reflects the differential regulatory preparation of male and female pathways. A synthesis of the regulatory modules implicated in this process, integrating transcriptomic meta-analyses and recent gene-specific evidence in *Prunus*, is summarized in Fig. 2.

## Future perspectives

Reproductive development in temperate fruit trees cannot be fully captured by a single dormancy–growth continuum governed by the fulfilment of chilling and heat requirements. This limitation arises because chilling and heat accumulation implicitly assume that temperature acts on a single continuous developmental programme. However, the evidence discussed here indicates that different reproductive processes respond to temperature in distinct seasonal windows. Under this condition, developmental outcome depends less on the total amount of cold or warmth received than on the order in which these thermal phases occur. Consequently, altered thermal sequencing becomes a more informative descriptor of reproductive failure than cumulative temperature deficits alone.

Within this interpretation, reproductive progression is organized through the sequential engagement of temperature-dependent processes. Male reproductive development advances during winter through the acquisition of meiotic competence and, in some species, the completion of microsporogenesis, whereas pollen maturation and female gametophyte development remain dependent on the transition to warmer spring temperatures.

Winter therefore cannot be regarded as a phase of generalized developmental arrest. Instead, it represents a period during which specific reproductive events are selectively enabled. Cold and warmth do not act as interchangeable signals across the reproductive programme but impose a temporal structure in which different processes respond within distinct thermal windows. This organization explains why key reproductive steps may advance in the absence of visible growth, why male and female gametophytes differ in thermal sensitivity, and why fertility is especially vulnerable to shifts in the timing and sequence of seasonal temperature cues rather than to their cumulative amounts alone.

Although the biphasic framework is supported by convergent anatomical, cytological, and transcriptomic evidence, it remains largely inferential. Much of the available support is based on temporal associations between seasonal temperature patterns and developmental transitions, rather than on direct experimental demonstration of causality. This limitation is not specific to the present model but reflects a broader constraint in the study of phenology and reproductive development in woody perennials, where temperature effects are most often inferred from field correlations or comparative seasonal datasets rather than from controlled perturbations (Chuine *et al*., 2016).

In most cases, coordinated shifts in gene expression, chromatin state, and developmental progression are described under contrasting winter or pre-bloom conditions without experimental manipulation sufficient to determine whether temperature acts as a primary developmental driver or as a permissive signal operating through upstream regulatory constraints. Meta-analyses of transcriptomic datasets in *Prunus* species identify recurrent seasonal patterns in the expression of meiotic and dormancy-related genes, yet these patterns remain correlative and do not resolve the causal hierarchy linking temperature perception, regulatory activation, and developmental outcome (Canton *et al*., 2021; Calle *et al*., 2022). As a result, the biphasic model still lacks the experimental resolution required to define the specific checkpoints at which thermal cues are interpreted by male and female developmental programmes.

A first requirement for strengthening the biphasic framework is to define the effective thermal input experienced by reproductive tissues within the bud. Most phenological and developmental interpretations continue to rely on air temperature as a proxy, despite buds being structurally complex organs composed of multiple tissue layers with distinct thermal, metabolic, and regulatory properties. Insulation by bud scales and radiative balance may further modulate the internal physiological environment of sporogenous tissues. Direct measurements at the bud surface have shown that the thermal environment experienced by buds can diverge substantially from air temperature and more accurately predict developmental transitions, highlighting the need to move from atmospheric proxies to organ-scale thermal sensing (Kleinknecht *et al*., 2015). Without direct measurements of internal bud temperature and its spatial heterogeneity, it remains uncertain how closely climatic records reflect the thermal signals integrated by male and female gametophyte development.

Beyond temperature perception itself, spatial heterogeneity raises a second, closely related issue that directly affects how the biphasic framework is evaluated: the scale at which reproductive development is analysed. Most molecular evidence for temperature-dependent regulation (transcriptomic, epigenetic, and chromatin based) has been generated from bulk samples, typically entire buds or composite tissues that include multiple cell types and developmental domains (Canton *et al*., 2021; Calle *et al*., 2022). This analytical scale contrasts with the core assumption of the biphasic model, which posits developmentally distinct modules differing in timing, competence, and thermal sensitivity. Structural features of the bud reinforce this limitation. Changes in vascular differentiation during dormancy progression indicate that physiological connectivity between the bud and the supporting shoot is dynamic rather than static (Bartolini *et al*., 2006; Andreini *et al*., 2012), implying that the delivery of mobile signals and resources varies across time and tissue domains. At the same time, studies in peach flower buds suggest that callose deposition differs between vegetative and reproductive contexts and may reflect tissue differentiation rather than uniform symplastic isolation (Joseph *et al*., 2026).

Taken together, these observations indicate that overwintering buds contain different tissues and reproductive structures that remain only partially connected, within which temperature-derived signals may be transmitted, buffered, or perceived differently. Under these conditions, reliance on whole-bud molecular datasets represents a conceptual limitation rather than a purely technical one. Bulk transcriptomic or epigenomic profiles inevitably average signals originating from tissues that are asynchronous in developmental state and thermal responsiveness, potentially masking the checkpoints that define cold- and heat-dependent phases. The feasibility of reducing this complexity has nevertheless been demonstrated. Laser capture microdissection has been used to isolate high-quality RNA from discrete meristematic domains of apple vegetative buds, demonstrating that spatially resolved molecular analyses are technically achievable (Verma *et al*., 2019). More recently, single-bud expression analyses have revealed pronounced transcriptional heterogeneity within peach buds during the cold season, indicating that whole-bud profiles obscure distinct regulatory states associated with dormancy progression (Puertes *et al*., 2023). These studies remain largely descriptive and have not yet been applied to explicitly test developmental modularity or thermal responsiveness at the level implied by the biphasic framework. Their contribution is therefore conceptual, exposing the gap between the spatial resolution of current datasets and the level of organization at which reproductive development is regulated.

A further limitation of the current evidence base is the scarcity of functional validation. Most evidence for temperature-driven regulation of reproductive development in temperate fruit trees is derived from robust temporal correlations linking chilling or warming regimes to gene expression dynamics, chromatin state, and cytological progression. Only a limited number of studies have moved beyond correlation to directly manipulate candidate regulators. Functional analyses of *DAM* genes provide rare but instructive examples. In apple, transgenic lines overexpressing or silencing *MdDAM4* demonstrated that this gene actively controls the timing of dormancy induction and release, with overexpression leading to earlier dormancy onset and delayed release, and silencing producing the opposite phenotype (Lempe *et al*., 2024). Similarly, heterologous overexpression of *PpeDAM6* in plum resulted in marked alterations of vegetative growth and hormone homeostasis, linking *DAM* activity to growth repression and dormancy maintenance through direct physiological effects (Lloret *et al*., 2021). Functional perturbation has also been reported outside the *DAM* family: in sweet cherry, overexpression of *PavTCP17* in Arabidopsis inhibited lateral bud outgrowth and altered ABA-related pathways, supporting a regulatory role inferred from cold-responsive expression patterns (Wen *et al*., 2023).

Despite their significance, these studies remain isolated. For most genes commonly invoked in dormancy and reproductive regulation—including meiotic regulators, DAM/SVP modules, hormone-related pathways, and stress-responsive transcription factors—it remains unclear whether they act as primary drivers of developmental transitions or represent downstream readouts of broader temperature-controlled physiological states. This gap underscores the need for functional approaches designed to test causality, including targeted genetic manipulation, transient assays, and controlled environmental perturbations, if biphasic models of reproductive development are to progress beyond descriptive synthesis.

Bridging these gaps is essential if the biphasic framework is to acquire predictive value. If male and female reproductive pathways are governed by distinct thermal windows and checkpoints, the model should anticipate when reproductive coordination is most likely to fail, which phases are intrinsically more vulnerable, and which species or cultivars are disproportionately exposed under altered seasonal patterns.

The strength of the biphasic model will therefore depend not on the identification of additional regulatory components, but on the ability to test its predictions across contrasting genotypes, environments, and thermal scenarios. Its value lies in restoring temporal structure to reproductive complexity. Whether this structure can be operationally validated will depend on our capacity to measure what is currently inferred, to disentangle what is currently averaged, and to manipulate—rather than merely observe—the developmental processes linking seasonal temperature to fertility. Under increasingly unstable climates, this distinction will be central not only to understanding how reproduction proceeds, but to explaining why it fails.

## Data Availability

This review contains no new experimental data.
